# Prevalence of bacteriocin genes in *Lactobacillus* strains isolated from fecal samples of healthy individuals and their inhibitory effect against foodborne pathogens

**DOI:** 10.22038/ijbms.2021.53299.11998

**Published:** 2021-08

**Authors:** Atieh Darbandi, Roya Ghanavati, Arezoo Asadi, Shiva Mirklantari, Meysam Hasannejad-Bibalan, Vahid Lohrasbi, Behrooz Sadeghi Kalani, Mahdi Rohani, Malihe Talebi, Mohammad Reza Pourshafie

**Affiliations:** 1 Department of Microbiology, School of Medicine, Iran University of Medical Sciences, Tehran, Iran; 2 Microbial Biotechnology Research Centre, Iran University of Medical Sciences, Tehran, Iran; 3 Behbahan Faculty of Medical Science, Behbahan, Iran; 4 Department of Microbiology, School of Medicine, Guilan University of Medical Sciences, Rasht, Iran; 5 Department of Microbiology, Faculty of Medicine, Ilam University of Medical Sciences, Ilam, Iran; 6 Clinical Microbiology Research Center, Ilam University of Medical Sciences, Ilam, Iran; 7 Department of Microbiology, Pasteur Institute of Iran, Tehran, Iran

**Keywords:** Bacteriocin, Caco-2 cells, Escherichia coli, Foodborne disease, Gene expression, Lactobacillus plantarum

## Abstract

**Objective(s):**

Foodborne diseases are considered as an important public health issue. The purpose of the current study was to isolate *Lactobacillus *spp. strains from fecal samples, investigate their antimicrobial properties, and assess the expression of genes encoding bacteriocin in co-culture of *Lactobacillus* with enteric pathogens.

**Materials and Methods:**

Fecal samples of healthy people were collected. Human colon adenocarcinoma cell line Caco-2 was used to examine *Lactobacillus *strains adherence capacity. Quantitative real-time reverse transcription PCR (qRT-PCR) was used to determine bacteriocin-encoding genes expression in co-culture of the selected *Lactobacillus *strain with *Salmonella, Shigella*, and two diarrheagenic *Escherichia coli *serotypes during 4, 6, and 24 hr of incubation.

**Results:**

The selected *L. plantarum *strain was able to inhibit four foodborne pathogens in both methods. *L. plantarum* No.14 exhibited the highest ability to adhere to Caco-2 cells. In this study, *pln F, sak P, pln I, pln B*, and *pln* J genes of *L. plantarum* No.14 were upregulated in co-culture of *L. plantarum* No.14 with diarrheagenic *E. coli *serotypes. In addition, acd, Lactacin F, sak P, pln J, pln EF, and pln NC8 genes as well as *pln NC8* and *pln A* genes mRNA levels were significantly increased in co-culture of *L. plantarum* No.14 with *Shigella dysenteriae,* and *Salmonella typhi,* respectively, during 24 hrs of incubation.

**Conclusion:**

Other studied genes were down-regulated during the incubation time. The selected *L. plantarum* strains could be served as alternative antimicrobial agents against pathogens which could contaminate foodstuffs and are responsible for human diseases.

## Introduction

Foodborne diseases are considered as an important public health problem and a significant impediment to socioeconomic development worldwide (1, 2). Many foodborne diseases may be self-limiting, but in some circumstances could reach life-threatening levels. Most of which are infections caused by a variety of bacteria, viruses, and parasites (3). The number of foodborne pathogenic bacteria included in Enterobacteriaceae family members. They could be transmitted through oral-fecal route. Some genera of this family causes of diarrhea, followed by sepsis, meningitis, and many enteric diseases (4). According to the WHO estimates of Global Burden of Foodborne Diseases, diarrheagenic *Escherichia coli* causes more than 300 million diseases and about 200,000 deaths worldwide annually (5). Inability to ensure food safety is one of the major food-related problems. Although chemical preservatives prevent the growth of harmful microorganisms, their use in the food industry is still unsafe for human health and the environment (3). 

Humans become infected by *Salmonella* through the consumption of food contaminated with animal feces. Because many mild cases are not diagnosed or reported, the exact number of infections may be thirty or more times greater than that reported. Center for Disease Control and Prevention (CDC) has estimated that *Salmonella* bacteria are the leading cause of 1.2 million illnesses, 23,000 hospitalizations, and 450 deaths in the United States every year. CDC data for the US show that annually, over 40,000 salmonellosis cases arise with 500 cases terminating towards death (6). *Shigella* bacteria could also be transmitted through eating contaminated food and drinking or swimming in contaminated water. Every year in the United States, *Shigella *bacteria cause about 500,000 diseases, 6000 hospitalizations, and 40 deaths. In the United States, 25,000 shigellosis cases are reported every year, while some estimates are as high as 450,000 cases per year; therefore, some cases are probably seriously under-reported (7).

In recent years, there has been an interest in the use of probiotics in the food industry as an alternative to antibiotics to prevent and control diseases (8). Several strains of Lactic acid bacteria (LAB), which are common inhabitants of human intestine, have been identified as probiotics.  In order to be considered as a probiotic, a microbe must possess a variety of characteristics recommended by FAO/WHO (9, 10). *Lactobacillus* spp. are members of LAB, which could exert a strong antagonistic activity against many microorganisms as a result of the production of antimicrobial agents, including organic acids, hydrogen peroxide, diacetyl, inhibitory enzymes, and bacteriocins (11, 12). Bacteriocins are a kind of ribosomal synthesized antimicrobial peptides secreted by some bacteria against microorganisms that are usually closely related to the producer microorganism; bacteriocins do not harm the producer bacteria due to the production of some specific immunity proteins (7, 13). Bacteriocins are divided into four distinct categories. Class I bacteriocins are modified peptides (lantibiotics) such as linear and globular peptides, which are active through the formation of pores, efflux of small metabolites from sensitive cells, or through enzyme inhibition. Class II bacteriocins are unmodified peptides (non-lanthionine), which are active by inducing membrane permeability and subsequent leakage of target bacteria molecules. Class II bacteriocins are subdivided into five subclasses including Class IIa (pediocin PA-1 like bacteriocins), Class IIb (composed of two peptides), Class IIc (circular peptides), Class IId (linear, non-pediocin-like, single-peptide bacteriocins), and Class IIe (linear, non-pediocin-like, three or four-peptide bacteriocins). Class III bacteriocins are large, heat-unstable proteins which are divided into two subclasses, including bacteriolytic (IIIa) and non-lytic peptides (IIIb), and Class IV bacteriocins are post-translationally modified circular peptides (14, 15). Competition of *Lactobacillus* spp. with pathogenic microorganisms, including *Salmonella*, *Shigella*, *E. coli*, and *Staphylococcus aureus*, makes LAB as attractive microorganisms for combating gastrointestinal illnesses and bacterial foodborne diseases. The purpose of the current study was to focus on bacteriocin genes of *Lactobacillus* strains isolated from fecal samples of healthy individuals and to evaluate the expression of genes encoding *L. plantarum* bacteriocin in co-culture with enteropathogenic microorganisms. In this study, the inhibitory effect of *L. plantarum* on the growth of pathogenic strains was evaluated by co-incubation of *Salmonella*,* Shigella,* and two diarrheagenic *E. coli* serotypes with the selected *Lactobacillus *strain. Also, the expression of genes related to the bacteriocin production was checked out by quantitative real-time reverse transcription PCR (qRT-PCR) reactions.

## Materials and Methods

***Bacterial isolates and growth conditions***

An experimental study was conducted. The isolates were collected from the fecal samples of healthy individuals who had received no antibiotic therapy over the past 6 months prior to sampling and did not have GI disorders at the sampling time in Tehran, Iran (14). The samples were plated on Man, Rogosa, and Sharpe agar (MRS) (Merck, Germany) and incubated for 48 hrs at 37 °C. Selected colonies were kept at -80 °C in MRS broth containing 20% glycerol.

***Molecular detection of lactobacillus isolates***

Total bacterial genomic DNA was extracted by Genomic DNA mini kit (Roche, Germany) to identify the genus of the isolated strains.* Lactobacillus* genus was identified by means of PCR method using 16S rRNA specific For-Lac (5´-TGGAAACAGGTGCTAATACCG-3´) and Rev-Lac (5´-CCATTGTGGAAGATTCCC-3´) primers and DNA purification kit (Roche, Germany).

PCR conditions were as follows: an initial denaturation step at 94 °C for 5 min, followed by 30 cycles of denaturation at 94 °C for 30 sec and 55 °C for 30 sec, an extension at 72 °C for 30 sec, and a final extension step at 72 °C for 7 min (16). The identification of isolated *Lactobacillus* species was performed using Multiplex PCR  assay as described previously (17) (Table 1). 

***Identification of bacteriocin-related genes***

The presence of 18 bacteriocin-related genes was investigated in 50 *Lactobacillus* isolates by PCR reaction using specific primers. PCR primers and annealing temperatures are presented in Table 1. PCR conditions were similar for all genes: an initial denaturation step at 94 °C for 5 min, followed by 34 cycles of denaturation at 94 °C for 30 sec, an extension at 72 °C for 60 sec, and a final extension at 72 °C for 10 min (18). The PCR products were identified by electrophoresis on 1.5% agarose gels stained with GelRed. One *Lactobacillus *strain with the highest frequency of bacteriocin encoding genes was selected to be examined for the presence of probiotic properties such as the ability to resist simulated gastrointestinal conditions, to hydrolyze bile salts, to inhibit biofilm formation by pathogens, and to attach to CT-26 human cancer cells.

***Screening for acidic pH and bile resistance***

The selected *L. plantarum* strain was initially tested for its ability to resist acidic pH and bile salts as described previously (19). Briefly, the selected* L. plantarum *strain was grown overnight in MRS broth. The cultures were then centrifuged at 6000 rpm, and the pellets were washed with phosphate buffered saline (PBS), buffer (pH 7.4). An initial count (CO) of the bacterial suspension was performed after a serial dilution (10−2 to 10−10) and plating on MRS agar, followed by incubating plates at 37 °C for 48 hr in anaerobic conditions. Then bacterial pellets were re-suspended in 5 ml of PBS buffer and incubated at 37 °C for 3 hrs while adjusting pH at 3.0 using 0.5 M HCl (Merck, Germany). 

Screening for bile-resistant isolates was performed by resuspension of the culture’s pellets in MRS broth containing 0.4% bile salts (Merck, Germany), followed by incubation for 6 hrs at the above mentioned conditions. The harvested bacterial biomass from PBS at pH 3.0 was washed in PBS, and the bacterial cells were enumerated as described above. The viable cells were grouped as strongly resistant, resistant, intermediate, and susceptible based on 2, 2-4, 4-6, and >6 log reduction in their counts, in comparison with the initial suspension, after 3 and 6 hrs of incubation in acid and bile, respectively (19).

***In vitro biofilm formation assay***

The selected* L. plantarum  *isolate was subjected to biofilm formation assay as described previously (20) with some modifications. For biofilm formation, wells of a 96-well plate were filled with 200 μl of 3×10^7^ CFU/ml bacteria under study and incubated without shaking for 72 hrs at 37 °C. The wells were washed with PBS, and the remaining attached bacteria were stained for 30 min with 200 μl of 0.1% (wt/vol) crystal violet in an isopropanol-methanol-PBS solution (1:1:18 [vol/vol/vol]). Excess stain was washed with 200 μl of water per well. Wells were air dried for 30 min, and the bounded dye was extracted with 200 μl of ethanol-acetone (80:20) solvent. The optical density (OD) of each well (135 μl) was measured at 570 nm using a microplate reader. The sterile medium and *Pseudomonas aeruginosa* ATCC 27853 were included as negative and positive controls, respectively.

***Screening for antibacterial activity***

***Agar spot tes*** *t*

Primary examination of the selected* L. plantarum* antimicrobial activity against the four most common foodborne pathogens, including enteropathogenic *E. coli* (EPEC) (ATCC 4388), enteroaggregative *E. coli* (EAEC), *S. typhi *(ATCC 19430), and *S. dysenteriae* (PTCC 1188) (obtained from Pasteur Institute of Iran and were not type strains), was performed by agar spot test according to Hernández *et al*. (2005) (21). Briefly, the strain was cultured in 5 ml of MRS broth at 37 ° C for 18 hrs, then 3 μl aliquots of overnight cultures of *L. plantarum* (No.14) isolates were spotted on MRS agar. After 18 hrs of incubation at 37 °C, the plate was overlaid with 5 ml of Trypticase soy broth (TSB) (Merck, Germany) or Brain heart infusion (BHI) (Merck, Germany) soft agar (containing 0.1% agar, w/v) and inoculated with the pathogenic strains and incubated at 37 °C for 24-72 hrs.

***Well-diffusion method***

In order to examine the nature of the inhibition mechanism, well-diffusion test was exploited according to Toba *et al*. (1991) (22). Briefly, 10 ml of BHI agar was mixed with 500 μl of an overnight culture of the pathogenic strains at 45 °C and poured into a petri dish. The selected* L. plantarum *strain was cultured in 5 ml of MRS broth (pH=6.5) at 37 °C for 18 hrs and then centrifuged (8000 × g for 20 min, 4 °C). The cell-free supernatant (CFS) was recovered and filter-sterilized through a 0.22 nm pore membrane. In order to rule out the possible inhibitory effects of organic acids, CFSs were neutralized with 2 M NaOH (pH=6.5). The supernatant was inserted into the wells (3 mm in diameter) in an agar layer. Eventually, after 24 hrs of incubation at 37 °C, the inhibition zone diameter wider than 6 mm was considered as positive.

***Attachment to caco-2 cells***

Caco-2 cell line (NCBI code: C466) was used in the adhesion assay. About 3 ml of 1.5×10^5^ cells/ml caco-2 cells were seeded on a 6-well plate containing RPMI (without antibiotics) (Gibco, Carlsbad, CA, USA) supplemented with 10% (v/v) fetal calf serum (Gibco, Life Technology, USA) and then incubated at 37 °C and 5% CO_2_. The culture medium was changed every other day and maintained for at least four days until reaching 70–90 % conﬂuency. One ml of the selected* L. plantarum *(10^8^ CFU. ml^−1^) and 1 ml of RPMI medium without antibiotics were added to each well, and the plate was incubated at 37 °C and 5 % CO_2_ for 2 hrs. For releasing unbound bacteria, the wells were washed four times with sterile PBS (23). Then 1 ml of methanol was added to each well at room temperature for 5-10 min to fix the cells. Gram staining was done, and excess dye was washed with distilled water. The wells were examined under oil immersion microscope with a magnification of 100X. 

***Co-culture of L. plantarum (No.14)*** ***with EPEC, EAEC, S. typhi, and S. dysenteriae***

The interference of the selected* L. plantarum *with the growth of pathogenic strains was evaluated by co-incubation with* S. typhi*, *S. dysenteriae*, EPEC, and EAEC, separately. Briefly, for each experiment, a suspension containing 5 ml of MRS broth and 5 ml of TSA broth was incubated with 10^5 ^CFU/ml of the selected* L. plantarum,* and then enteric pathogen was added to each tube. All tubes were incubated at 37 °C for 4, 6, and 24 hrs. After incubation, bacterial pellet was collected by centrifugation at 5000 ×g for 15 min, re-suspended in PBS, and followed by vortex mixing to disrupt all aggregates (24).

***Expression of genes related to bacteriocin production***

The expression of bacteriocin-encoding genes in the selected* L. plantarum *(*gaaA*,* pln A, pln B, pln EF,* *Lactacin F, pln G, plnI, plnJ, pln NC8, sak P, *and* acd) *was determined in co-culture with *S. typhi*, *S. dysenteriae*, EPEC, and EAEC using qPCR. qPCR primers and annealing temperatures are shown in supplementary data in Table 1.

Total RNA was isolated from bacterial pellet using a total RNA extraction kit (Roche, Germany) according to the manufacturer’s instructions. RNA was quantified by absorbance at OD_260/280_, and only samples with a ratio of 1.8±2.0 were used for cDNA synthesis. RNA integrity was determined by running each sample on a denaturing 2% agarose gel. Prime Script cDNA synthesis kit (Takara Bio, Japan) was used for synthesis of cDNA. cDNA samples were stored at -20 °C until required for further investigations.

qPCR was performed in a Corbett rotor-gene 6000 detection system using RealQ Plus Master Mix Green (Ampliqon, Denmark). The cDNA content of each target gene was normalized to internal standard 16S mRNA. The serially diluted pooled cDNA samples were used as templates to generate a standard curve between the CT values of each gene and logarithm of cDNA template concentrations. The standard curves were served as positive controls for qPCR and suggested similar priming efficiency between each target gene and *16s rRNA *gene. The reactions using water as template were served as negative controls for qPCR. The relative expression levels of target genes were analyzed using a comparative threshold cycle (2^−ΔΔCt^) method (25). Real-time PCR reactions were complemented by agarose gel electrophoresis, melting curve analysis, and confirmation of primer specificity and synthesis cDNA”

***Statistical analysis***

Errors in experimental data of mean values were expressed by the Microsoft Excel 2010 program as standard deviations. Statistical differences in multiple groups were determined using one-way ANOVA test. Data were considered as statistically significant with *P* <.05 (*P<.05, **P<.01, ***P<.001, and ****P<.0001).

## Results

***Identification of fecal isolates***

Ultimately, out of 60 fecal samples, 50 isolates were identified as *Lactobacillus *species based on 16S rRNA gene amplification. The frequency of *Lactobacillus *species was reported as follows: *L. plantarum,* 30 (50%); *L. casei,* 10 (16.6%); *L. rhamnosus*, 6 (10%); *L. brevis*, 2 (3.3%); *L. delbruecki, *1 (1.6%); and *L. fermentum,* 1 (1.6%). It is worth noting that sample sequencing confirmed the results of molecular analysis. Among the 60 healthy fecal samples, only 30 isolates (50%) were identified as *L. plantarum*.

***Identification of bacteriocin genes***

**Figure 1:**
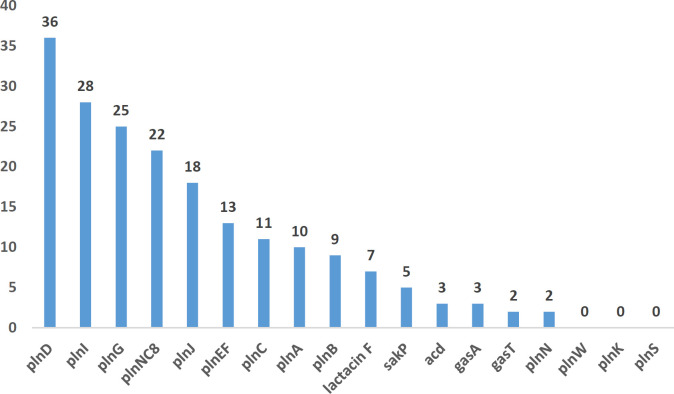
Bacteriocin-related genes detection. Most Bacteriocin-related genes are plnD, plnI and plnG, respectively

All *Lactobacillus* species were tested for the presence of bacteriocin genes; the frequency of which was reported as follows: Plantaricin K, 0; Plantaricin I, 28(56%); Plantaricin S, 0; Plantaricin G, 25(50%); Plantaricin EF,13 (26%); Plantaricin A, 10 (20%); Plantaricin B, 9 (18%); Plantaricin N, 2 (4%); Plantaricin C, 11 (22%); Plantaricin W, 0; Gassericin A, 3 (6%); Gassericin T, 2 (4%); Sakacin P, 5 (10%); Plantaricin NC8, 22 (44%); Lactacin F, 7 (14%); Plantaricin J, 18 (36%); Plantaricin D, 36 (72%); and Acidocin, 3 (6%) (Figure 1). According to the results, 7 different *Lactobacillus* species had the ability to produce bacteriocin. The most bacteriocin-producing* Lactobacillus *species was *L. plantarum* (77%), and the least bacteriocin-producing species was reported to be *L. delbruecki* (5.5%). One of the *L. plantarum *isolates (*L. plantarum* No.14) was positive for the presence of 11 bacteriocin genes. This *L. plantarum* (No.14) strain with the highest frequency of bacteriocin-encoding genes was selected for further studies.

***In vitro biofilm formation and screening for pH and bile resistance***

The ratio of biofilm formation was calculated by measuring the observed optical density (OD) of the *L. plantarum* (No.14) strain in comparison to *P. aeruginosa* as a positive control. The results showed that *L. plantarum* (No.14) was significantly stronger than positive control in biofilm formation (*P*<0.05). In addition, *L. plantarum* (No.14) was able to resist low pH (i.e. pH 3.00) and 0.4% bile salts.

***Screening for antibacterial*** ***activity***

*L. plantarum* (No.14) was screened for the potential bacteriocin production using the agar spot test and well diffusion method. *L. plantarum* (No.14) showed high inhibitory activity against the pathogenic bacteria (Table 2).

***Cell line and adhesion assay***

*L. plantarum* (No.14) ability to adhere to Caco-2 cells was examined microscopically using a light microscope. It was strongly adhesive (270 attached bacterial cells /20 microscopic fields) and exhibited the highest ability to adhere to Caco-2 cells.

***Quantitative real-time PCR***

**Figure 2:**
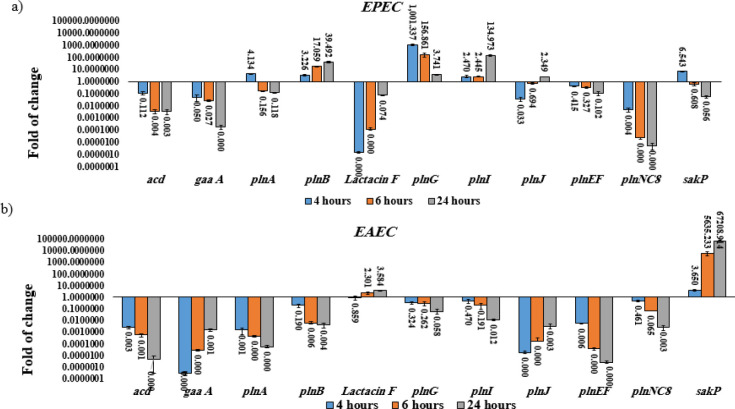
Relative fold change (relative to the culture of *L. plantarum* alone) in bacteriocin genes expression in co-culture of *L. plantarum *with: a) enteropathogenic *Escherichia coli* (EPEC), A significant downregulation of *acd, gaa A, pln F, pln EF,* and *pln* *NC8* mRNA levels (*P* value < .05) and upregulation of *pln B, pln I, *and *pln J* mRNA levels (*P *value <.05) were showed in a time-dependent manner, as compared to *L. plantarum *(No.14) culture alone; b) enteroaggregative *E. coli *(EAEC), A significant downregulation of *acd, gaa A, pln A, pln, pln G, pln I, pln J, pln EF*, and *pln* NC8 and upregulation of Lactacin F and sak P mRNA levels were showed in a time-dependent manner, as compared to* L. plantarum* (No.14) culture alone. Data were normalized with 16s rRNA gene of *L. plantarum*. Data were represented as mean ±SD

Since *L. plantarum* (No.14) had the highest frequency of bacteriocin-encoding genes as well as the inhibitory activity and the ability to adhere to Caco-2 cells, the expression of bacteriocin-encoding genes in this strain was investigated in co-culture with the gut pathogens using qPCR method. Bacteriocin genes expression was investigated in co-culture of *L. plantarum* (No.14) with EPEC, EAEC, *S. dysenteriae, *and *S. typhi*, separately at different incubation times. Co-culture of *L. plantarum* (No.14) with EPEC resulted in a significant downregulation of *acd* (~0.11 to ~0.00), *gaa A* (~0.05 to ~0.00), *pln F* (~0.00 to ~0.07), *pln EF* (~0.41 to ~0.10), and *pln NC8* (~0.00 to ~ 0.00) mRNA levels (*p* value < .05) and up-regulation of *pln B *(~3.22 to ~39.49)*, pln I *(~2.46 to ~134.97), and* pln J* (~0.03 to ~2.34) mRNA levels (*P* value <.05) in a time-dependent manner, as compared to *L. plantarum* (No.14) culture alone. Also, *pln A *(~4.13 to ~0.11)*, pln G* (~1001.33 to ~3.74), and *sak P* (~6.54 to ~0.05) genes were upregulated significantly in the first 4 hours of co-culture (Figure 2a). But after 4 hours, the expression of these genes gradually decreased (*P* value < .05) (Figure 1, Supplementary data).

As shown in Figure 2b, co-culture of *L. plantarum* (No.14) with EAEC significantly reduced* acd* (~0.00 to ~0.00E), *gaa A* (~2.81E to ~0.00),* pln A* (~0.00 to ~0.00)*, pln B* (~0.19 to ~0.00)*, pln G* (~0.32 to ~0.05)*, pln I* (~0.47 to ~0.01)*, pln J* (~0.00E to ~0.00)*, pln EF *(~0.00 to ~0.00E), and* pln NC8* (~0.46 to ~0.00) genes expression in a time-dependent manner. On the other hand, prolonged co-culture significantly increased *Lactacin F *(~0.85 to ~3.58) and *sak P* (~3.64 to ~67208.91) genes expression, simultaneously (*p *value <.05) in a time-dependent manner (Figure 2, Supplementary data).

**Figure 3:**
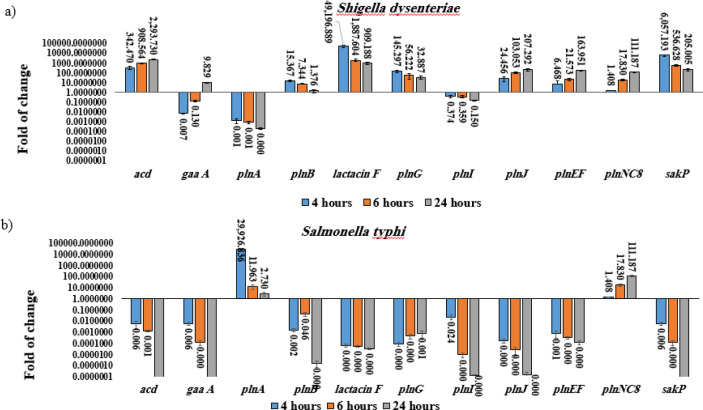
Relative fold change (relative to the culture of *Lactobacillus plantarum *alone) in bacteriocin genes expression in co-culture of *L. plantarum *with: a) *Shigella dysenteriae*, a significant downregulation of *gaa A, pln, and pln I *mRNA levels and up-regulation of *acd, Lactacin F, sak P, plnJ, pln EF,* and *pln* NC8 genes were showed in a time-dependent manner, as compared to* L. plantarum* (No.14) culture alone (*P*<0.05); b) *Salmonella typhi*, a significant downregulation of *sak P, acd, gaa A, pln B, Lactacin F, pln G, pln I, pln J*, and *pln* EF mRNA levels and upregulated *pln NC8* and *pln A* mRNA levels (*P*< .05) in a time-dependent manner as compared to *L. plantarum *(No.14) culture alone. Data were normalized with *16s rRNA* gene of *L. plantarum*. Data were represented as mean ±SD

Co-culture of *L. plantarum* (No.14) with *S. dysentriae *resulted in a significant downregulation of *gaa A *(~0.130 to ~0.007), *pln A *(~0.001 to ~0.000), and *pln I* (~0.374 to ~0.150) genes mRNA levels after 4 and 6 hrs, while the expression of *acd *(~342.47 to ~2.293.730), *Lactacin F *(~909.188 to ~49.196.889), *sak P *(~205.005 to ~6.057.193), *plnJ* (~24.456 to ~207.292), *pln EF* (~6.468 to ~163.951), and *pln NC8* (~1.408 to ~111.187) genes was significantly increased compared to the other genes (*P*<.05)(Figure 3a)(Figure 3, Supplementary data).

As shown in Figure 3b, co-culture of *L. plantarum* (No.14) with *S. typhi* significantly reduced *sak P *(~0.006 to ~0.00)*, acd *(~0.006 to ~0.00), *gaa A *(~0.006 to ~0.00), *pln B *(~0.064 to ~0.00), *Lactacin F *(~0.00 to ~0.00), *pln G *(~0.0061 to ~0.00), *pln I *(~0.024 to ~0.00), *pln J *(~0.00 to ~0.00), and *pln EF *(~0.001 to ~0.00) genes expression and up-regulated *pln NC8 *(~1.408 to ~111.187) and *pln A* (~2.730 to ~29.926.83) genes mRNA levels (*P*< .05) in a time-dependent manner (Figure 4, Supplementary data).

**Table 1:** Bacteriocin-related primer sequences used in this study

Reference	TM	Amplicon Size(bp)	Sequence (5′–3′)	Primer	Bacteriocin Gene
(47)	51	102	GACCACAGCGAACATT AATGAGGCACCAGAAG	*GaAF* *GaAR*	**Gassericin A**
(48)	52	84	GTTGCAGGATCATGTG TGTTGCAGCTCCGTTA	*LFAF* *LFAR*	**Acidocin**
(49)	61	320	GCC TTA CCA GCG TAA TGC CC CTG GTG ATG CAA TCG TTA GTT T	*plnF* *PlnR*	**Plantaricin S**
(50)	56	710	GGTGGGAGAAATAATTGGGC CCTATTACAAACGATATGGCC	*GaTF* *GaTR*	**Gassericin T**
(18)	57	184	AGTCGTTGTTGGTGGAAGAAAT TCTTATCTTGCCAAAACCACCT	*LaFF* *LaFR*	**Lactase F**
(45)	59	217	GGT CTG CGT ATA AGC ATC GC AAA TTG AAC ATA TGG GTG CTT TAA ATT CC	*PlnF* *PlnR*	**PlantaricinNC8 structural gene**
(51)	54	165	TCA CAC GAA ATA TTC CA GGC AAG CGT AAG AAA TAA ATG AG	*PlnF* *PlnR*	**PlantaricinW structural gene**
(52)	49	451	GTA CAG TAC TAA TGG GAG CTT ACG CCA ATC TAT ACG	*PlnAF* *PlnAR*	**Plantaricin A**
(53)	56	170	TTC AGA GCA AGC CTA AAT GAC GCC ACT GTA ACA CCA TGA C	*PlnBF* *PlnBR*	**Plantaricin B**
(54)	56	971	AGC AGA TGA AAT TCG GCA G ATA ATC CAA CGG TGC AAT CC	*PlnCF* *PlnCR*	**Plantaricin C**
(53)	57	415	TGA GGA CAA ACA GAC TGG AC GCA TCG GAA AAA TTG CGG ATA C	*PlnDF* *PlnDR*	**Plantaricin D**
(55)	56	428	GGC ATA GTT AAA ATT CCC CCC CAG GTT GCC GCA AAA AAA G	*PlnEFF* *PlnEFR*	**Plantaricin EF**
(53)	59	450	CTC GAC GGT GAA ATT AGG TGT AAG CGT TTA TCC TAT CCT CTA AGC ATT GG	*PlnIF* *PlnIR*	**Plantaricin I**
(55)	54	475	TAA CGA CGG ATT GCT CTG AAT CAA GGA ATT ATC ACA TTA GTC	*PlnJF* *PlnJR*	**Plantaricin J**
(55)	55	246	CTG TAA GCA TTG CTA ACC AAT C ACT GCT GAC GCT GAA AAG	*PlnKF* *PlnKR*	**Plantaricin K**
(53)	56	454	TGC GGT TAT CAG TAT GTC AAA G CCT CGA AAC AAT TTC CCC C	*PlnGF* *PlnGR*	**Plantaricin G**
(53)	57	146	ATT GCC GGG TTA GGT ATC G CCT AAA CCA TGC CAT GCA C	*PlnNF* *PlnNR*	**Plantaricin N**
(56)	60	126	GGAGTAGGTGGAGCGACAGT TCCACCAGTAGCTGCCGTTA	*SaF* *SaR*	**Sakacin P**

**Table 2:** Determination of *Lactobacillus plantarum *antibacterial activity

Indicator Bacteria	Agar Spot Test N (%)	Well- Diffusion Method N (%)
Enteroaggregative *E. coli* (EAEC)	15.46 ± 1*.*01	17*.*83 ± 0*.*66
Enteropathogenic *E.coli* (EPEC)	18.32 ± 0*.*4	16.75 ± 0*.*31
*S. dysentriae*	14.12 ± 0*.*9	13.35 ± 1*.*4
*S. typhi*	12.91 ± 1.51	11.52 ± 0*.*8

## Discussion

The outbreaks of foodborne diseases occur daily in all countries and have become as important social topics. In the United States, foodborne pathogens cause an estimated 48 million illnesses annually (26, 27). Approximately, 179 million acute gastroenteritis (AGE) cases occur in the United States each year (28). There is an urgent need to develop a methodology for the reduction of these pathogens (29). In this study, *Lactobacillus *was shown to have antibacterial effects against a number of foodborne bacteria, including *EPEC*, *EAEC*, *S. typhi, *and *S. dysentriae.* *L. plantarum* strains were identified as the most common isolates in this study. *L. plantarum* strains are more resistant to the GI tract microbiota in comparison with other LAB strains. They have been extensively employed as probiotic in the food industry (30). New strains intended to be used as probiotic must be sufficiently characterized according to the FAO/WHO and EFSA guidelines (9). One of the most important criteria that should be considered in selecting probiotic strains is to have specific functional characteristics (4). It should be noted that from a total of 60 healthy individuals’ fecal samples, 50 bacteriocin-producing *Lactobacillus* strains were isolated. In this project, most bacteriocins were reported to be Plantaricin D (36, %72), Plantaricin I (28, %56), and Plantaricin G (25, %50), respectively. In this study, 7 different *Lactobacillus* species had the ability to produce bacteriocin. *L. plantarum *and* L. casei* were the most bacteriocin-producing *Lactobacillus *species (77%). Surprisingly, it was found that *L. plantarum* was positive for the presence of 11 bacteriocin genes, and 15 (50%) *L. plantarum* strains were able to inhibit four foodborne pathogens in both methods. Therefore, for the above reasons, *L. plantarum* was chosen for further studies. Also, *L. plantarum* No.14 exhibited the highest ability to adhere to Caco-2 cells. Many studies have reported that* Lactobacillus *strains of intestinal flora origin significantly inhibited the growth of diarrheagenic *E. coli *pathotypes (31-34). Another critical characteristic that should be considered in choosing novel probiotic isolates is their ability to colonize and survive in the gastrointestinal tract (30). Since the wide diversity in the microbial population of the human gut could justify the presence of different *L. plantarum *strains with different biological activities, such variation in* Lactobacillus* strains adherence capacity was expected.* L. plantarum* strain No. 14 showed high ratios of these two abilities *in vitro*, indicating that this strain could efficiently colonize the intestine and exert its antimicrobial activity. In this study, the expression of bacteriocin-encoding genes in *L. plantarum* No.14 was investigated in co-culture with different pathogens using qPCR method. Bacteriocins are easily degraded by digestive and proteolytic enzymes, while remaining active in the food substrate in which they are located (35). Therefore, they are used in the food industry to preserve food through suppressing spoilage organisms and foodborne pathogens, which cause foodborne illnesses or food spoilage. In addition, they are non-immunogenic, safe, and non-toxic (36). A significant up-regulation was detected in *pln I* (134.97 fold), *pln B* (39.49 fold), and *pln J* (2.34 fold) genes as well as in *pln F* (3.58 fold) and* sak P* (67208.91) genes mRNA levels after co-incubation of *L. plantarum *No.14 with EPEC and EAEC, respectively, during the co-incubation time (*P*<.05). A significant up-regulation was also detected in *acd* (2.293.730 fold), *pln F* (40.196.88), sak P (6.057.193), *pln G* (145.297), *pln J* (207.292), and *pln EF *(163.951) genes as well as in *pln NC8(111.187)* and *pln A* (29.926.83) genes mRNA levels after co-incubation of *L. plantarum *No.14 with* S. dysentriae* and *S. typhi*, respectively, in a time-dependent manner (*P*< .05). The factors affecting the growth of bacteriocin-producing LAB strains or the ability to produce bacteriocin may be strain-dependent and could even vary depending on the different types of bacteriocins. The difference in the antimicrobial mechanisms may be imputed to the versatility in molecular structure, gene expression, or bacterial strains, resulting in diversity in acid tolerance, hydrogen peroxide tolerance, and sensitivity to bacteriocins (37, 38). In this study, co-culture of *L. plantarum *No.14 with four pathogens resulted in various profiles of bacteriocin-related genes expression. This finding suggests that under specific environmental conditions, metabolites and structural features of specific strains of microorganisms which are exposed to *L. plantarum,* may play a role in determining the expression of bacteriocin-associated gene loci (39). *L. plantarum* coordinately could express several types of bacteriocins depending on the environmental conditions, which could possibly be associated with the bacteriocin regulatory system (40). The production of several bacteriocins by the same strain plays an important role in ecological niche competition as each bacteriocin may possess a special inhibitory mechanism, thereby widening the spectrum of inhibited bacteria (41, 42). Other studied genes (*acd, gaaA,* *plnA, plnEF, plnNC8, *and* plnG*) were down-regulated during the incubation time, suggesting that non-bacteriocin-producing mutants might have been selected during sub-culturing. On the other hand, all *pln *operons are repressed under nonproducing conditions. In addition, environmental factors such as small variations in culture conditions (media composition, temperature, pH) and the presence of a dense microbial population (quorum) affect the synthesis of bacteriocins, particularly those bacteriocins that are regulated by quorum-sensing and may be strain-specific (41, 43). Bacteriocin genes are often located on mobile genetic elements such as transposons and plasmids. Therefore, bacteriocins genes could enter into pathogenic bacteria via conjugation and develop bacteriocin-resistant variants (7, 44). In this study, *L. plantarum* produced several types of bacteriocins in co-culture with four pathogens, coordinately. This is very important because a high concentration of various bacteriocins was suddenly produced at a certain time, preventing the emergence of bacteriocin-resistant variants (41, 45, 46). Thus, these findings support the hypothesis that studied *L. plantarum* strains could be served as natural antimicrobial agents against pathogens which could contaminate foodstuffs and are responsible for human diseases.

## Conclusion

The results of this study highlighted the antibacterial potency of *L. plantarum* No.14 of human origin against foodborne pathogens. Co-culture of *L. plantarum* No.14 with EPEC, EAEC, *S. dysentriae, *and* S. typhi* exhibited high levels of bacteriocin-related genes expression. The selected *L. plantarum* strains could be used as bio-preservative agents or supplements to prevent bacterial gastrointestinal infections after identifying their active component, testing their cytotoxic effects, and validating their safety *in vitro* and *in vivo* models.
